# A Vector-Based Motion Retargeting Approach for Exoskeletons with Shoulder Girdle Mechanism

**DOI:** 10.3390/biomimetics10050312

**Published:** 2025-05-12

**Authors:** Jiajia Wang, Shuo Pei, Junlong Guo, Mingsong Bao, Yufeng Yao

**Affiliations:** 1 State Key Laboratory of Robotics and System, Harbin Institute of Technology, Harbin 150001, China; hitw0423@163.com (J.W.); peishuohit@163.com (S.P.); 2 Department of Mechanical Engineering, School of Naval Architecture and Ocean Engineering, Harbin Institute of Technology (Weihai), Weihai 264200, China; 3 Shandong Guoxing Smartech Co., Ltd., Qingdao 365500, China; msbao@suprobot.com

**Keywords:** upper limb exoskeletons, motion retargeting, vector methods, multiple motion representation methods

## Abstract

Shoulder girdle plays a dominant role in coordinating the natural movements of the upper arm. Inverse kinematics, optimization, and data-driven approaches are usually used to conduct motion retargeting. However, these methods do not consider shoulder girdle movement. When the kinematic structure of human and that of exoskeletons share a similar joint configuration, analytical motion retargeting methods can be used for exoskeletons with shoulder girdle mechanism. This paper proposes a vector-based analytical motion retargeting approach for exoskeletons with shoulder girdle mechanism. The approach maps the vectors of the upper limb segments to the joint space using vector-based methods. Simulation results using four different motion descriptions confirm the method’s accuracy and efficiency.

## 1. Introduction

Exoskeletons are rapidly advancing technologies with applications in rehabilitation [1,2,3,4], teleoperation [5,6,7,8], and human augmentation [9,10,11]. Generating natural, efficient, and dynamically stable human-like motions is crucial, but remains a fundamental challenge due to differences in kinematics, dynamics, and actuation constraints between the human and the robotic system.

Motion retargeting, the process of transferring human motion to a different embodiment, such as an exoskeleton, is an effective method for generating human-like motion. Traditionally, motion retargeting is performed by manually defining a mapping between two different morphologies (e.g., a human actor and an exoskeleton), using methods based on Inverse kinematics (IK) and optimization techniques. IK methods can be categorized into differential IK and analytical IK. Marcia et al. employed an iterative differential approach to map hierarchical human feature points to robot joints [12]. Dariush et al. extended this approach by incorporating joint limit constraints and self-collision avoidance penalties to generate physically feasible robot motion [13]. For Spherical-Rotational-Spherical (SRS) structured robots, analytical IK can provide efficient closed-form solutions for mapping the human end-effector pose to a robotic arm, enabling motion retargeting [14,15,16]. However, mapping only the end-effector pose may lead to significant differences in intermediate joints, such as the elbow. Yang et al. addressed this issue by proposing an anthropomorphic motion retargeting framework that introduces the normalized normal vector of the arm plane to determine the elbow position [17]. Optimization-based methods model motion retargeting as a constrained optimization problem to ensure the generated motion respects the robot’s physical limits. Suleiman et al. formulated an optimization problem that considers joint limits, torque constraints, and dynamic feasibility, and solved it recursively using a dynamics-based optimization algorithm [18]. Similarly, Tairan He et al. minimized joint position differences between human and humanoid robots using the Adam optimizer to implement motion retargeting [5]. However, these iterative methods suffer from high computational costs and are not suitable for real-time applications.

On the other hand, data-driven motion retargeting has been used to circumvent the manual mapping process by leveraging machine learning methods. Such learning based methods enjoy flexibility and scalability as they reduce the need for excessive domain knowledge and tedious tuning processes required to define pose features properly. Owing to the merits of such methods, a human pose can be mapped to a exoskeletons pose by employing a statistical model called shared Gaussian process latent variable models (GPLVM) [19]. Many data-driven techniques have relied on GPLVM to construct shared latent spaces between the two motion domains [20,21,22]. Hang et al. proposed associate latent encoding (ALE), which uses two different variational auto-encoders (VAEs) with a single shared latent space [23]. A similar idea was extended in [24] by incorporating negative examples to aid safety constraints. However, one clear drawback of data-driven motion retargeting method is the need to gather a sufficiently large dataset beforehand.

Most existing IK-based, optimization-based, and data-driven methods focus on human arms, but lack support for shoulder girdle movement. The natural movements of the upper arm are strongly coordinated with the movements of the shoulder girdle, represented by the scapulohumeral rhythm (SHR). However, few studies have incorporated shoulder girdle kinematics in their retargeting frameworks, limiting their applicability to achieve a full range of upper-limb movements. In addition, when the kinematic structure of human and that of exoskeletons share a similar joint configuration, analytical method can provide computational efficiency and high accuracy. Moreover, different studies adopt specific motion representation methods, such as joint positions [25]; end-effector poses; and kinematic parameters including shoulder girdle angles, swivel angle, and wrist position [26]. Our research focuses on developing a unified approach to map these diverse representations into the joint space of exoskeletons.

In this paper, a vector-based analytical motion retargeting approach for exoskeletons with shoulder girdle mechanism is proposed. Our method leverages vector-based kinematic transformations to achieve efficient and accurate motion retargeting, eliminating the computational burden of optimization-based methods and the heavy data dependency of data-driven approaches. Moreover, the proposed method supports multiple input motion representations, enabling its applicability to a wide range of tasks. The contributions of this paper are as follows:

- A vector-based analytical motion retargeting approach is proposed for exoskeletons with shoulder girdle mechanism, mapping the vectors of the upper limb segments to the joint space through a vector-based method with high computational efficiency and precision.
- The approach can accommodate four motion representation methods: (a) joint positions; (b) the end-effector (wrist) pose; (c) shoulder girdle angles, swivel angle, and wrist position (SGASAWP); and (d) polynomial descriptions of the SHR, swivel angle, and wrist position (SHRSAWP).

The remainder of this paper is organized as follows: Section 2 summarizes the kinematic structure of the upper limb exoskeleton. Section 3 presents the vector-based analytical motion retargeting approach. Section 4 discusses how different motion representation methods can be mapped into the joint space using the approach. Section 5 verifies the effectiveness and universality of the proposed approach through simulation experiments. Finally, Section 6 presents conclusions and outlines future work.

## 2. The Kinematic Structure of the Upper Limb Exoskeleton

The human upper limb consists of multiple joints with a wide range of motion and flexibility. However, multiple joints present challenges in determining the degrees of freedom (DOF) arrangement for exoskeletons. The shoulder girdle alone comprises four joints [27], making it inefficient and unnecessary to replicate each joint’s motion individually in an exoskeleton. The movement of the shoulder girdle contributes significantly to the translational motion of the glenohumeral (GH) joint, particularly during elevation–depression and protraction–retraction. Studies have shown that the trajectory of the GH joint follows two circular arcs, with the distance between the axes of rotation deviating by less than 3 mm [28]. Based on this observation, the exoskeleton can use two orthogonal rotational joints to support the movement of the shoulder girdle. For the GH joint, which is a ball-and-socket joint, the exoskeleton can employ three intersecting rotational joints to mimic its motion. The elbow joint, which supports forearm flexion and extension, can be modeled as a single revolute joint with its axis perpendicular to the plane defined by the upper arm and forearm.

**Figure 1:**
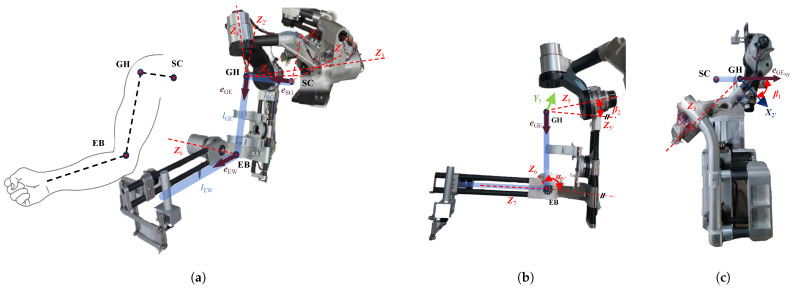
The configuration of FREE II. (**a**) Mapping between human and FREE II. (**b**) Detailed view of upper arm link. (**c**) Top view of FREE II.

The exoskeleton FREE II is a six-DOF exoskeleton with a shoulder girdle mechanism utilizing parallelograms [29]. The configuration of FREE II is illustrated in Figure 1. The axis $Z_{2^{\prime }}$ which is parallel to $Z_{2}$ but rotates in the opposite direction, has been added to model the kinematics of the parallelogram mechanism. To represent the Denavit–Hartenberg (DH) parameters *d* and *a* solely using the lengths of upper arm and forearm, axis $Z_{5^{\prime }}$ is also added. The DH parameters for FREE II are listed in Table 1 [30].

The FK of FREE II can be derived as follows:

(1) Tend0 =T2110T2′2T32′T43T54T5′5T65′Tend6T

where

(2) Tii−1=cosθi−sinθi0ai−1sinθicosαi−1cosθicosαi−1−sinαi−1−sinαi−1disinθisinαi−1cosθisinαi−1cosαi−1cosαi−1di0001

(3) T5′5=cosβ20sinβ200100−sinβ20cosβ200001

## 3. The Vector-Based Analytical Motion Retargeting Approach

Owing to the alignment of the anatomical and robotic joint axes, along with the rigid human–robot connection, the orientations of the joints and links in the exoskeleton remain fixed relative to the vectors of the upper limb segments of the wearer. Consequently, given the vectors of the upper limb, the joint orientations of the exoskeleton can be determined, thereby defining the configuration of the upper limb exoskeleton. The vectors of the shoulder girdle, upper arm, and forearm are represented by $e_{\mathrm{SG}}$, $e_{\mathrm{GE}}$, and $e_{\mathrm{EW}}$, respectively. The relationships between the orientations of joints and links in the exoskeleton and $e_{\mathrm{SG}}$, $e_{\mathrm{GE}}$, and $e_{\mathrm{EW}}$ are as follows:

The vectors of the upper arm and forearm links in exoskeleton are parallel to the wearer’s upper arm and forearm, respectively, i.e.,: $e_{\mathrm{GE}}=X_{5^{\prime }}$, $e_{\mathrm{EW}}=Y_{6}=Z_{7}$.

The elbow joint axis must be perpendicular to the vectors of upper arm and forearm. The orientation of elbow joint is determined by:

(4) $$Z_{6}=\frac{e_{\mathrm{GE}}\times e_{\mathrm{EW}}}{∥e_{\mathrm{GE}}\times e_{\mathrm{EW}}∥}$$

Given two non-parallel vectors located on the upper arm link in the exoskeleton: $Z_{6}$ and $X_{5^{\prime }}$, the posture of the upper arm link is uniquely determined. As illustrated in Figure 1b, the vectors $Z_{5}$ and $Z_{5^{\prime }}$, both located on the upper arm link, can be calculated using the following expressions:

(5) $$Z_{5^{\prime }}=Rot\left(e_{\mathrm{GE}},-\alpha_{5^{\prime }}\right)Z_{6}$$

(6) $$Z_{5}=Rot\left(Y_{5^{\prime }},-\beta_{2}\right)Z_{5^{\prime }}$$

where $Y_{5^{\prime }}=Z_{5^{\prime }}\times X_{5^{\prime }}$.

The axis of the second joint $Z_{2}$ is perpendicular to the vector of shoulder girdle $e_{\mathrm{SG}}$ and $Z_{1}$. Thus, $Z_{2}$ is determined by:

(7) $$Z_{2}=Z_{2^{\prime }}=\frac{e_{\mathrm{SG}}\times Z_{1}}{∥e_{\mathrm{SG}}\times Z_{1}∥}$$

where $Z_{2^{\prime }}$ is parallel with $Z_{2}$, $Z_{0}=Z_{1}={\left[0\;0\;1\right]}^{T}$.

The axis of the third joint $Z_{3}$ remains fixed relative to $e_{{\mathrm{SG}}_{xy}}$, which is the unit vector of $e_{\mathrm{SG}}$ projected onto the XY-plane, due to the presence of the parallelogram mechanism. Therefore, $Z_{3}$ can be determined by:

(8) $$Z_{3}=Rot\left(X_{2^{\prime }},\alpha_{3}\right)Z_{2^{\prime }}$$

where $X_{2^{\prime }}=Rot\left(Z_{2},\beta_{1}\right)e_{{\mathrm{SG}}_{xy}}$.

Given that the links are rigid bodies, the angles between $Z_{4}$ and $Z_{3}$, as well as between $Z_{4}$ and $Z_{5}$ remain constant. Therefore, $Z_{4}$ can be determined using Equation (9):

(9) $$\left\{\begin{array}{c} Z_{3}\cdot Z_{4}=c\alpha_{3}\\ Z_{4}\cdot Z_{5}=c\alpha_{4}\\ ∥Z_{4}∥=1 \end{array}\right.$$

**Figure 2:**
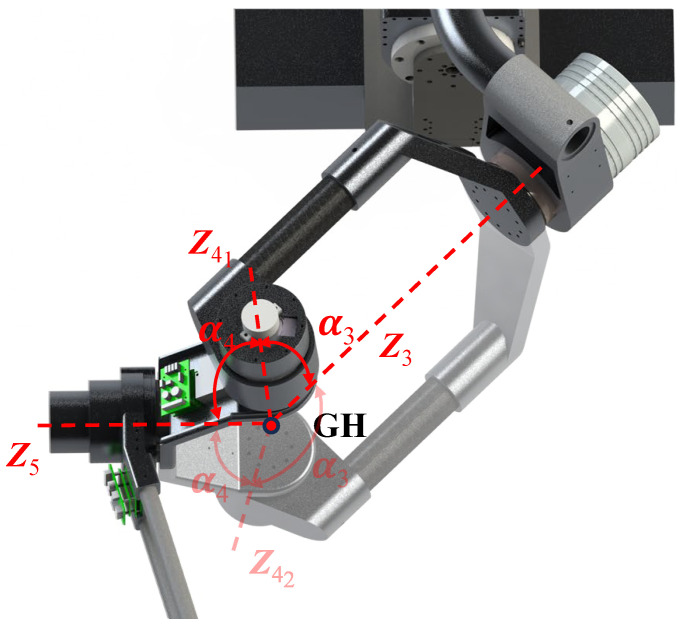
Diagram of two solutions of $Z_{4}$. The angle between $Z_{4}$ and $Z_{3}$ is $\alpha_{3}$, and the angle between $Z_{4}$ and $Z_{5}$ is $\alpha_{4}$.

By expressing $z_{4x}$ and $z_{4y}$ in terms of $z_{4z}$ using the two equations from the Equation (9), and then substituting them into the remaining equation, the system is reduced to a quadratic equation in $z_{4z}$, which can then be solved for $Z_{4}$. Diagram of two possible solutions of $Z_{4}$ is shown in Figure 2.

According to the DH criterion, after obtaining $Z_{i}$, the $X_{i}$ can be obtained, which is perpendicular to the plane formed by $Z_{i}$ and $Z_{i+1}$:

(10) $$X_{i}=\mathrm{sign}\left(\alpha_{i}\right)\frac{Z_{i}\times Z_{i+1}}{∥Z_{i}\times Z_{i+1}∥}$$

where $i=2^{\prime },3,4$.

To obtain $X_{5}$, $X_{5^{\prime }}$ is rotated around $Y_{5^{\prime }}$ by an angle $-\beta_{2}$, as follows:

(11) $$X_{5}=Rot\left(Y_{5^{\prime }},-\beta_{2}\right)\;X_{5^{\prime }}$$

Then $\theta_{i}$ is the angle between $X_{i-1}$ and $X_{i}$:

(12) $$\left|\theta_{i}\right|=\mathrm{acos}\left(X_{i-1}\cdot X_{i}\right)$$

where i=3,4,5,6.

## 4. Mapping Different Motion Representation Methods into the Joint Space Using the Approach

### 4.1. Joint Positions

When the motion representation method is joint (GH, elbow, and wrist) positions, the vectors of the shoulder girdle $e_{\mathrm{SG}}$, upper arm $e_{\mathrm{GE}}$, and forearm $e_{\mathrm{EW}}$ can be obtained by:

(13) $$\begin{array}{cc} e_{\mathrm{SG}}= & \frac{P_{\mathrm{GH}}}{∥P_{\mathrm{GH}}∥}\\ e_{\mathrm{GE}}= & \frac{P_{\mathrm{EB}}-P_{\mathrm{GH}}}{∥P_{\mathrm{EB}}-P_{\mathrm{GH}}∥}\\ e_{\mathrm{EW}}= & \frac{P_{w}-P_{\mathrm{EB}}}{∥P_{w}-P_{\mathrm{EB}}∥} \end{array}$$

Then $\theta_{i}$ can be determined using the proposed approach in Section 3.

### 4.2. End-Effector Pose

When the motion representation method is the end-effector pose, the vector of forearm can be determined as follows:

(14) $$e_{\mathrm{EW}}=Z_{\mathrm{end}}$$

where Zend=Tend0 (1:3,3).

**Figure 3:**
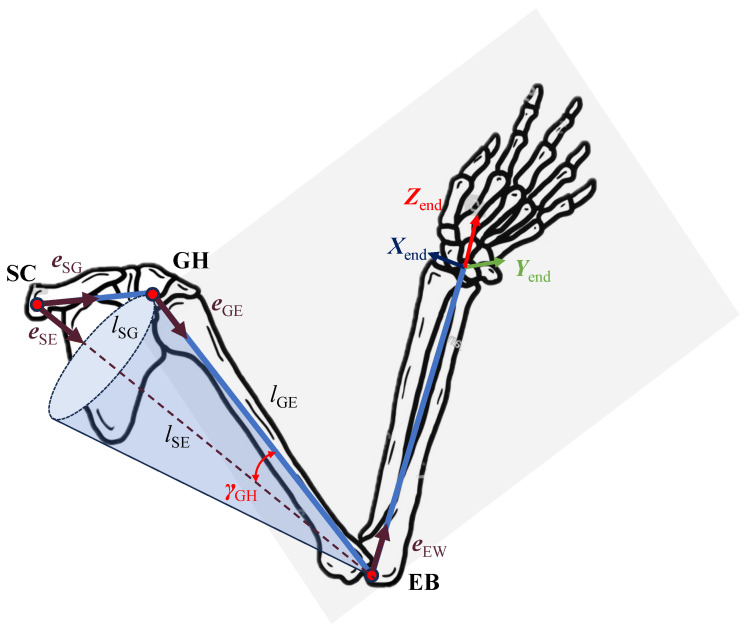
Constraint of the orientation of upper arm $e_{\mathrm{GE}}$. The angle between $e_{\mathrm{GE}}$ and $e_{\mathrm{SE}}$ is $\gamma_{\mathrm{GH}}$. And $Y_{\mathrm{end}}$ is perpendicular to $e_{\mathrm{GE}}$.

**Figure 4:**
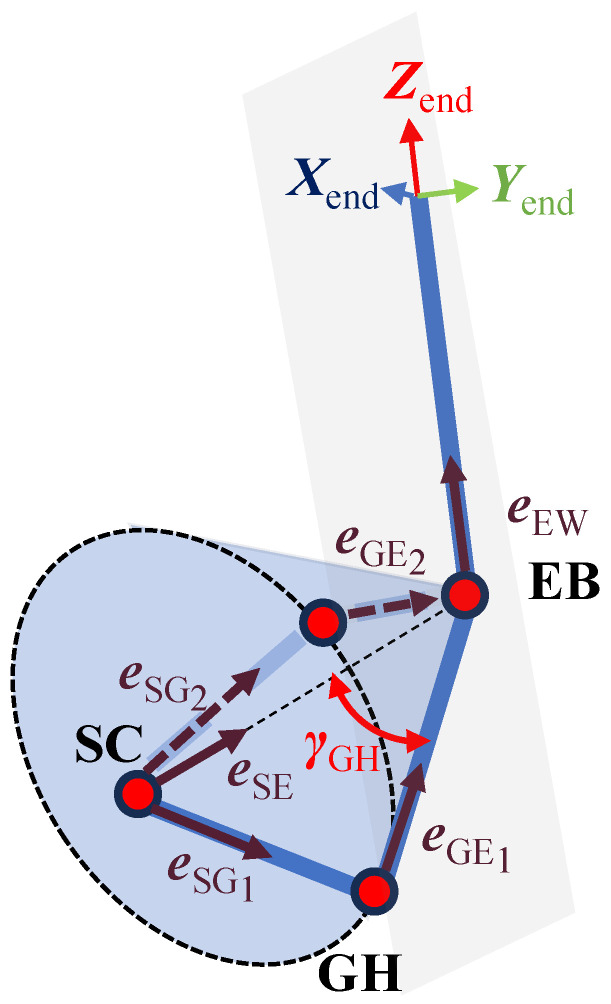
Two possible configurations of upper limb. The angle between $e_{\mathrm{GE}}$ and $e_{\mathrm{SE}}$ is $\gamma_{\mathrm{GH}}$, indicating that $e_{\mathrm{GE}}$ lies on the generatrix of a cone with $e_{\mathrm{SE}}$ as its axis and a half-angle of $\gamma_{\mathrm{GH}}$. Additionally, since $e_{\mathrm{GE}}$ must also lie within a plane perpendicular to $Y_{\mathrm{end}}$ (the gray plane), its possible positions are confined to the intersection of the cone and the plane, i.e., $e_{{\mathrm{GE}}_{1}}$ and $e_{{\mathrm{GE}}_{2}}$.

The upper arm, shoulder girdle and $e_{\mathrm{SE}}$ form a triangle and the angle between $e_{\mathrm{GE}}$ and $e_{\mathrm{SE}}$ is denoted as $\gamma_{\mathrm{GH}}$, as shown in Figure 3. $Y_{\mathrm{end}}$ is parallel to $Z_{6}$ and perpendicular to the plane formed by $e_{\mathrm{GE}}$ and $e_{\mathrm{EW}}$. $e_{\mathrm{GE}}$ can be solved using three constraints:

(15) $$\left\{\begin{array}{c} e_{\mathrm{GE}}\cdot e_{\mathrm{SE}}=c\gamma_{\mathrm{GH}}\\ e_{\mathrm{GE}}\cdot Y_{\mathrm{end}}=0\\ ∥e_{\mathrm{GE}}∥=1 \end{array}\right.$$

where $c\gamma_{\mathrm{GH}}=\cos\gamma_{\mathrm{GH}}=\frac{l_{\mathrm{SE}}^{2}+l_{\mathrm{GE}}^{2}-l_{\mathrm{SG}}^{2}}{2l_{\mathrm{SE}}l_{\mathrm{GE}}}$, $l_{\mathrm{SE}}=∥P_{W}-e_{\mathrm{EW}}l_{\mathrm{EW}}∥$, $e_{\mathrm{SE}}=\frac{P_{W}-e_{\mathrm{EW}}l_{\mathrm{EW}}}{l_{\mathrm{SE}}}$, PW=Tend0 (1:3,4), Yend=Tend0 (1:3,2). Diagram of the two solutions of $e_{\mathrm{GE}}$ is shown in Figure 4.

Vector of shoulder girdle can be obtained using $e_{\mathrm{GE}}$ and $e_{\mathrm{EW}}$:

(16) $$e_{\mathrm{SG}}=\frac{P_{W}-e_{\mathrm{GE}}\;l_{\mathrm{GE}}-e_{\mathrm{EW}}\;l_{\mathrm{EW}}}{l_{\mathrm{SG}}}$$

Finally, $\theta_{i}$ can be obtained using $e_{\mathrm{SG}}$, $e_{\mathrm{GE}}$, and $e_{\mathrm{EW}}$, as detailed in Section 3.

### 4.3. SGASAWP

When the motion representation method is SGASAWP, the vector $e_{\mathrm{SG}}$, $e_{\mathrm{GE}}$, and $e_{\mathrm{EW}}$ can be determined by calculating the joint position.

Given the shoulder girdle angles $\theta_{1}$ and $\theta_{2}$, the position of GH joint can be expressed as:

(17) $$P_{\mathrm{GH}}=\left[\begin{array}{c} p_{\mathrm{GH}x}\\ p_{\mathrm{GH}y}\\ p_{\mathrm{GH}z} \end{array}\right]=\left[\begin{array}{c} l_{\mathrm{SG}}c_{1}c_{2}\\ l_{\mathrm{SG}}s_{1}c_{2}\\ l_{\mathrm{SG}}s_{2} \end{array}\right]$$

where $c_{i}=\cos\theta_{i}$ and $s_{i}=\sin\theta_{i}$.

**Figure 5:**
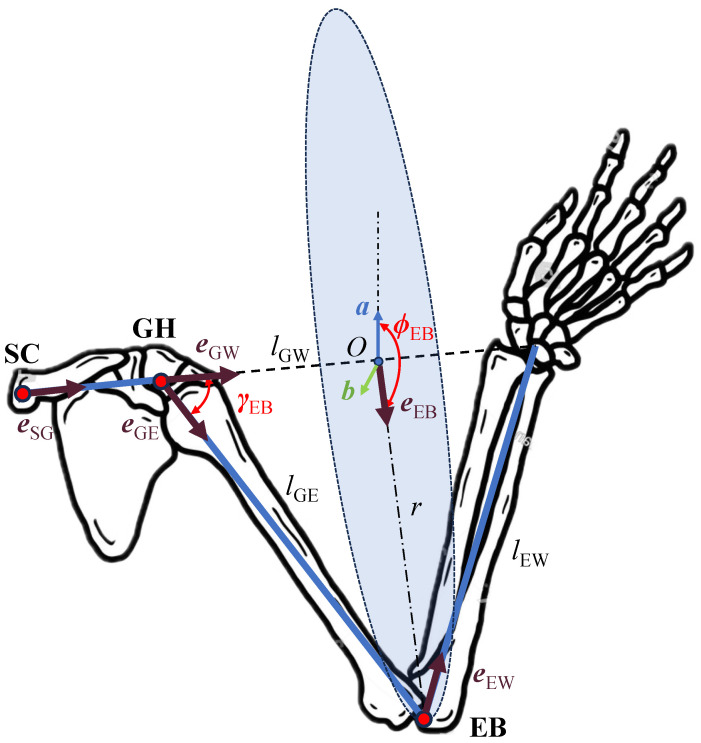
Diagram of the swivel angle. Given the position of GH and wrist, the set of possible $P_{\mathrm{EB}}$ is on the dashed circle. The swivel angle $\phi_{\mathrm{EB}}$ is the angle between a and $e_{\mathrm{EB}}$. $\gamma_{\mathrm{EB}}$ is the angle between $e_{\mathrm{GW}}$ and $e_{\mathrm{GE}}$.

The set of possible $P_{\mathrm{EB}}$ is on a circle, as shown in Figure 5. The circle radius is:

(18) $$r=l_{\mathrm{GE}}\sin\left(\gamma_{\mathrm{EB}}\right)=l_{\mathrm{GE}}\sqrt{1-\cos{\left(\gamma_{\mathrm{EB}}\right)}^{2}}$$

where $\cos\left(\gamma_{\mathrm{EB}}\right)=\frac{l_{\mathrm{GW}}^{2}+l_{\mathrm{GE}}^{2}-l_{\mathrm{EW}}^{2}}{2l_{\mathrm{GW}}l_{\mathrm{GE}}}$, $l_{\mathrm{GW}}=∥P_{W}-P_{\mathrm{GH}}∥$.

The circle center can be obtained using the following expression:

(19) $$O=P_{\mathrm{GH}}+l_{\mathrm{GE}}\cos\left(\gamma_{\mathrm{EB}}\right)e_{\mathrm{GW}}$$

where $e_{\mathrm{GW}}=\frac{P_{W}-P_{\mathrm{GH}}}{∥P_{W}-P_{\mathrm{GH}}∥}$.

Given swivel angle $\phi_{\mathrm{EB}}$, $P_{\mathrm{EB}}$ can be solved by:

(20) $$P_{\mathrm{EB}}=O+re_{\mathrm{EB}}$$

where $e_{\mathrm{EB}}=a\cos\left(\phi_{\mathrm{EB}}\right)+b\sin\left(\phi_{\mathrm{EB}}\right)$, $b=\frac{e_{\mathrm{GW}}\times \left(-Y\right)}{∥e_{\mathrm{GW}}\times \left(-Y\right)∥}$, and $a=b\times e_{\mathrm{GW}}$.

After obtaining $P_{\mathrm{GH}}$, $P_{\mathrm{EB}}$, and $P_{w}$, the vectors of the shoulder girdle $e_{\mathrm{SG}}$, upper arm $e_{\mathrm{GE}}$, and forearm $e_{\mathrm{EW}}$ can be solved by Equation (13), respectively. Finally, $\theta_{i}$ can be obtained as detailed in Section 3.

### 4.4. SHRSAWP

When the motion representation method is SHRSAWP, both $\theta_{1}$ and $\theta_{2}$ need to be determined from the feasible solutions to best match the SHR.

The polynomial fitting method is commonly used to model the coupled motion of the shoulder girdle with the elevation of the upper arm. One of these models was selected to validate our algorithm:

(21) $$\left\{\begin{array}{c} \bar{\theta_{1}}=4.34\times 10^{-5}\theta_{E}^{3}-3.21\times 10^{-3}\theta_{E}^{2}+0.1\theta_{E}-0.06\\ \bar{\theta_{2}}=-5.28\times 10^{-7}\theta_{E}^{4}+7\times 10^{-5}\theta_{E}^{3}-3.92\times 10^{-3}\theta_{E}^{2}\\ +0.04\theta_{E}+0.13 \end{array}\right.$$

where angle of elevation of the upper arm $\theta_{E}=\mathrm{acos}\left(\frac{p_{\mathrm{EB}y}-p_{\mathrm{GH}y}}{l_{\mathrm{GE}}}\right)$.

Both $\theta_{1}$ and $\theta_{2}$ can be determined by minimizing the object function:

(22) $$err\left(\theta_{1},\theta_{2}\right)=∥\theta_{1}-\bar{\theta_{1}}∥+∥\theta_{2}-\bar{\theta_{2}}∥$$

Then $\theta_{i}$ can be obtained as detailed in Section 4.3.

## 5. Numerical Simulation

This section focuses on the validation of the proposed approach. The FREE II was selected as the object of the simulation. The simulation results including joint positions, end-effector pose, SGASAWP and SHRSAWP are described in Section 5.1, Section 5.2, Section 5.3, and Section 5.4, respectively. The time consumption and source of errors are discussed in Section 5.5. The algorithm was run on a computer with an Intel i9-12900H processor and 16 GB RAM (Intel, Santa Clara, CA, USA).

### 5.1. Joint Positions

Firstly, the expected trajectories of $P_{\mathrm{GH}}$, $P_{\mathrm{EB}}$, and $P_{W}$ were obtained using a motion capture system (Mars2H, Nokov, Nosálov, Czech Republic, sampling rate: 60 Hz). These expected trajectories were then used to determine the IK solutions $\theta_{i}$ using the method described in Section 4.1. The determined IK solutions $\theta_{i}$ were finally substituted into FK to calculate the joint positions to compare with the expected ones. The resolution of the exoskeleton joints was considered to improve the realism of the simulation.

**Figure 6:**
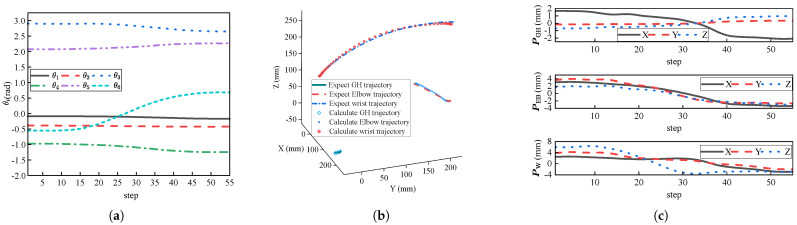
Simulation results when the motion representation method is joint positions. (**a**) One randomly selected set of IK solutions. (**b**) Trajectories of expected and calculated joint positions. (**c**) Error of joint positions.

When the motion representation method is joint positions, there are two different sets of IK solutions. One set of results is displayed in Figure 6. The corresponding joint angles are shown in Figure 6a. Figure 6b illustrates the expected and calculated trajectories of joint positions, while Figure 6c presents the associated errors. The link lengths of exoskeleton were fixed, whereas human upper limb segment lengths captured via motion capture system varied with soft tissue artifacts and marker displacement. This discrepancy will bring joint position errors, and the maximum mean position error could be captured within 4.7 mm.

The CLIK method employs a differential approach to map human feature points to robot joints, and introduces a closed-loop error term to minimize the Cartesian error. A comparison between our algorithm and the CLIK method is presented in Table 2. Compared to the CLIK method, our approach demonstrates superior accuracy and efficiency.

### 5.2. End-Effector Pose

Firstly, the expected end-effector pose trajectories were obtained by using a set of IK solutions from Section 5.1 as input for FK. The expected trajectories were then used to obtain IK solutions $\theta_{i}$ using the method described in Section 4.2. Finally, the calculated trajectories were determined by substituting the computed IK solutions $\theta_{i}$ into FK to compare with the expected ones.

**Figure 7:**
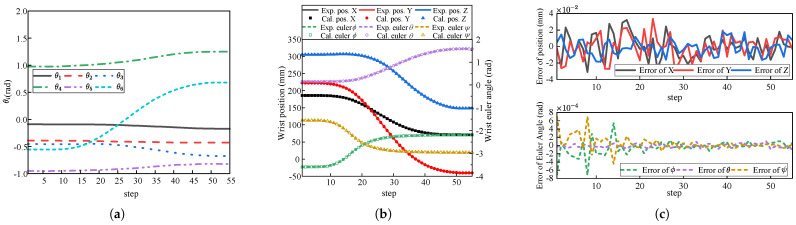
Simulation results when the motion representation method is the end-effector pose. (**a**) One randomly selected set of IK solutions. (**b**) Trajectories of expected and calculated end effector pose. (**c**) Error of position and Euler angle.

When the motion representation method is the end-effector pose, there are four different sets of IK solutions. A randomly selected set of results is displayed in Figure 7, where “EXP.” and “Cal.” in the legend stand for “expected” and “calculated”, respectively. The corresponding joint angles are shown in Figure 7a. Figure 7b illustrates the expected and calculated end-effector position and orientation, while Figure 7c presents the associated errors. The expected and calculated trajectories of wrist position and orientation aligned closely with each other. And the mean position error was approximately 0.018 mm and the mean Euler angle error was approximately $9.07\times 10^{-5}$ rad.

The comparison between our algorithm and the Jacobian-based method was shown in Table 3. Compared to the Jacobian-based method, the proposed approach using the vector method demonstrated superior performance in terms of solving speed and accuracy. Although the Jacobian-based method can improve accuracy by lowering threshold of error, its efficiency tends to decrease as a result.

### 5.3. SGASAWP

Firstly, the expected trajectories of shoulder girdle angles, swivel angle, and wrist positions were obtained by using a set of IK solutions from Section 5.1 as input for FK. The expected trajectories were then used to determine the IK solutions $\theta_{i}$ using the method described in Section 4.3. Finally, the calculated trajectories of the shoulder girdle angles, swivel angle, and wrist positions were determined by substituting the computed IK solutions $\theta_{i}$ into FK to compare with the expected ones.

**Figure 8:**
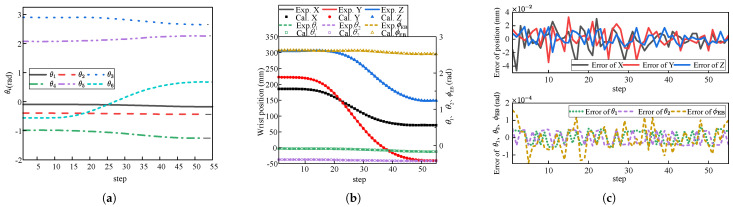
Simulation results when the motion representation method is SGASAWP. (**a**) One randomly selected set of IK solutions. (**b**) Trajectories of expected and calculated $\theta_{1}$, $\theta_{2}$, $\phi_{\mathrm{EB}}$, and $P_{W}$. (**c**) Error of $\theta_{1}$, $\theta_{2}$, $\phi_{\mathrm{EB}}$, and $P_{W}$.

When the motion representation method is SGASAWP, there are two different sets of IK solutions. A randomly selected set of results is shown in Figure 8. The corresponding joint angles are displayed in Figure 8a. Figure 8b illustrates the expected and calculated trajectories of shoulder girdle angles, swivel angle, and wrist positions, while Figure 8c presents the associated errors. The expected and calculated trajectories of the shoulder girdle angles, swivel angle, and wrist positions aligned closely with each other. The mean position error was about 0.017 mm and the mean swivel angle error was about $6.0\times 10^{-5}$ rad.

### 5.4. SHRSAWP

Firstly, the expected trajectories of swivel angle and wrist positions were obtained by using a set of IK solutions from Section 5.1 as input for FK. The expected trajectories were then used to determine the IK solutions $\theta_{i}$ using the method described in Section 4.4. The IK solutions $\theta_{i}$ were subsequently used to calculate the angle of elevation of the upper arm $\theta_{E}$ and the trajectories of the shoulder girdle angle, swivel angle, and wrist positions through FK. The expected shoulder girdle angles $\theta_{1}$ and $\theta_{2}$ were obtained using the polynomial representation of SHR. Finally, the calculated trajectories were compared with the expected ones.

**Figure 9:**
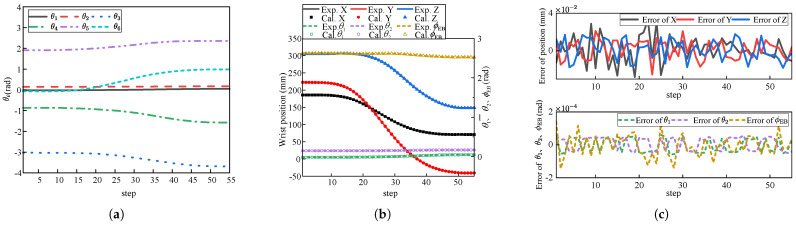
Simulation results when the motion representation method is SHRSAWP. (**a**) One randomly selected set of IK solutions. (**b**) Trajectories of expected and calculated $\theta_{1}$, $\theta_{2}$, $\phi_{\mathrm{EB}}$, and $P_{W}$. (**c**) Error of $\theta_{1}$, $\theta_{2}$, $\phi_{\mathrm{EB}}$, and $P_{W}$.

When the motion representation method is SHRSAWP, there are two different sets of IK solutions. A randomly selected set of these solutions is displayed in Figure 9. The corresponding joint angles are illustrated in Figure 9a. The expected and calculated trajectories of shoulder girdle angles, swivel angle, and wrist positions are shown in Figure 9b, while the associated errors are depicted in Figure 9c. The expected and calculated trajectories of the shoulder girdle angles, swivel angle, and wrist positions aligned closely with one another. The mean position error was approximately 0.018 mm, and the mean error in the shoulder girdle angles was approximately $4.50\times 10^{-4}$ and $4.19\times 10^{-5}$ rad. The comparison between our algorithm and GEAA method was shown in Table 4. Upper arm movements inherently induce shoulder girdle motion, and shoulder girdle motion in turn affects the upper arm motion. Therefore, the SHR in arm motion introduces an additional layer of complexity to motion retargeting. In the GEAA method, the relationship between the shoulder girdle angle and the upper arm elevation angle strictly follows predefined joint rhythms, and the upper arm configuration can be solved through optimization. In our approach, the shoulder girdle angles can be solved using an optimization process, while the upper arm configuration can be computed analytically. Motion retargeting can be achieved using our approach, while the SHR can also be maintained by minimizing the error of shoulder girdle joint angles. Compared to the GEAA, our algorithm achieved superior solving speed and greater accuracy due to reducing the number of optimization parameters and algebraic equations.

### 5.5. Discussion

The primary source of the proposed approach was the accumulated numerical error in solving the inverse trigonometric functions. The approach was implemented in MATLAB R2023b and C++ 2019. The calculation time of four motion representation methods was listed in Table 5. The algorithm’s computation time increased with the number of inverse solution sets. Compared to the joint position representation method, when the motion representation method was the end-effector pose, both the number of inverse solutions and the computation time nearly doubled. In the SGASAWP method, since the shoulder girdle angles were given, the computation time was shortest. Conversely, in the SHRSAWP method, where optimization algorithms were implemented, the longest computation time was limited to 15 ms. When the approach was implemented with C++ running on industrial PC CX6015-0100 (Beckhoff Automation Inc., Verl, Germany) under the software environment of TwinCAT3, the time consumption ranges from 0.0014 ms to 0.04 ms. Although m-file implementations ran slower by about one orders of magnitude, they can still be used for real-time control.

## 6. Conclusions

A vector-based analytical motion retargeting approach for exoskeletons with shoulder girdle mechanism was proposed. Given the vectors of upper limb segments, this approach leveraged the alignment of anatomical and robotic joint axes, along with the rigid human-robot connection, to determine the orientation of each exoskeleton joint. The approach could map four upper limb motion representation methods to the joint space of exoskeleton. The simulation results validated its accuracy and efficiency. Computational times for the four motion representation methods are 0.0145 ms, 0.0236 ms, 0.0127 ms, and 13.5 ms, respectively. When the motion representation was joint positions, the fixed link lengths of the exoskeleton did not match the varying lengths of human upper limb segments, and the positional error was relatively larger. For the other three representations, the maximum angular error and positional error can be captured within $4.5\times 10^{-4}$ rad and 0.018 mm, respectively.

Addressing the challenges associated with upper limb rehabilitation with full wrist freedom is the future research focus. Also, Kalman filter algorithm will be employed to mitigate the noise encountered during joint position acquisition.

## Figures and Tables

**Table 1:** DH parameters of FREE II.

*i*	$\alpha_{i-1}$	$a_{i-1}$	$d_{i}$	$\theta_{i}$
1	0	0	0	$\theta_{1}$
2	90	0	0	$\theta_{2}$
2′	0	$l_{\mathrm{SG}}$	0	$\beta_{1}-\theta_{2}$
3	86.47	0	0	$\theta_{3}$
4	−90	0	0	$\theta_{4}$
5	85	0	0	$\theta_{5}$
5′	Rotation with respect to $y_{5}$ by $\beta_{2}$			
6	−95	$l_{\mathrm{GE}}$	0	$\theta_{6}$
End	−90	0	$l_{\mathrm{EW}}$	0

**Table 2:** Comparison with CLIK when the motion representation method is joint positions.

Method	Mean Error of *P*_GH_ (mm)	Mean Error of *P*_EB_ (mm)	Mean Error of *P*_W_ (mm)	Time (ms)
Vector method	1.44	3.94	4.73	0.0145
CLIK [13]	3.86	4.49	6.90	0.1891

**Table 3:** Comparison with Jacobian-based method when the motion representation method is the end-effector pose.

Method	Mean Position Error (mm)	Mean Euler Angle Error (rad)	Calculation Time (ms)
Vector method	0.018	$9.07\times 10^{-5}$	0.0236
Jacobian-based method [31]	0.022	$1.11\times 10^{-4}$	1.3

**Table 4:** Comparison with GEAA method when the motion representation method is SHRSAWP.

Method	Mean Position Error (mm)	Mean Error of *θ*_1_ (rad)	Mean Error of *θ*_2_ (rad)	Calculation Time (ms)
Vector method	0.018	$4.50\times 10^{-4}$	$4.19\times 10^{-5}$	13.5
GEAA [26]	0.021	0.039	$6.41\times 10^{-5}$	27.5

**Table 5:** Calculation time of the four motion representation methods.

Representation Method	Joint Positions	End-Effector Pose	SGASAWP	SHRSAWP
m-file (ms)	0.0145	0.0236	0.0127	13.5
C++ (ms)	0.0015	0.0030	0.0014	0.04

## Data Availability

The research data are not publicly available as the study is ongoing. However, the data can be requested for academic reasons by contacting hitw0423@163.com.
